# Current Concepts in the Treatment of Retinitis Pigmentosa

**DOI:** 10.1155/2011/753547

**Published:** 2010-10-11

**Authors:** Maria A. Musarella, Ian M. MacDonald

**Affiliations:** ^1^Department of Ophthalmology, SUNY Downstate Medical Center, Brooklyn, NY 11203, USA; ^2^Department of Ophthalmology, Royal Alexandra Hospital, University of Alberta, 10240 Kingsway Avenue, Rm. 2319, Edmonton, Canada AB T5H 3V9

## Abstract

Inherited retinal degenerations, including retinitis pigmentosa (RP) and Leber congenital amaurosis (LCA), affect 1 in 4000 individuals in the general population. A majority of the genes which are mutated in these conditions are expressed in either photoreceptors or the retinal pigment epithelium (RPE). There is considerable variation in the clinical severity of these conditions; the most severe being autosomal recessive LCA, a heterogeneous retinal degenerative disease and the commonest cause of congenital blindness in children. Here, we discuss all the potential treatments that are now available for retinal degeneration. A number of therapeutic avenues are being explored based on our knowledge of the pathophysiology of retinal degeneration derived from research on animal models, including: gene therapy, antiapoptosis agents, neurotrophic factors, and dietary supplementation. Technological advances in retinal implant devices continue to provide the promise of vision for patients with end-stage disease.

## 1. Retinitis Pigmentosa

Retinitis pigmentosa (RP) describes a heterogeneous group of inherited retinal dystrophies characterized by progressive photoreceptor cell degeneration that affects approximately 1 in 4000 in a general population [1]. The genetics of RP is varied; nonsyndromic cases may be inherited as an autosomal dominant (30%), autosomal recessive (20%), X-linked recessive (15%), or sporadic/simplex traits (30%), and 5% may be early-onset and grouped as part of Leber congenital amaurosis [2]. Rarer forms also exist: X-linked dominant, mitochondrial, and digenic (due to mutations in two different genes). While RP is a disease usually limited to the eye, it may occur as part of a syndrome; as examples, Usher syndrome and Bardet-Biedl syndrome. Approximately 20%–30% of patients with RP have an associated nonocular disease and would be classified as having syndromic RP. A list of nonsyndromic and syndromic RP is maintained through RetNet (http://www.sph.uth.tmc.edu/retnet/). A majority of the genes associated with RP are expressed in either the photoreceptors or the retinal pigment epithelium (RPE). There is considerable variation in the severity of these conditions; the most severe being recessively inherited conditions generally resulting in loss of function of an important protein in a pathway.

RP is characterized by progressive degeneration of the retina usually starting in the midperiphery of the fundus and advancing towards the macula and fovea. The most common form of RP is a rod-cone dystrophy in which night blindness is the first symptom, followed by progressive loss of peripheral visual field. Classic clinical findings include: bone spicule pigmentation or pigment clumping, retinal arteriolar narrowing, waxy pallor of the optic nerve, epiretinal membrane formation, atrophy of the RPE and choriocapillaris (starting at the midperiphery of the retina with preservation of the RPE in the macula until late in the disease), posterior subcapsular cataract, epiretinal membrane formation, and cystoid macular edema (CME) [1].

Potentially important findings can be obtained from ERG recordings. The term rod-cone dystrophy, commonly used to describe RP, denotes the predominant system affected by retinal degeneration (rod versus cone) and is reflected by the rod-driven responses of the ERG being more severely affected than cone-driven responses. Early in the disease, the rod ERG amplitude is affected more than the cones; and with progression, the rod and cone responses are “extinguished”. Visual field testing often reveals a mid-peripheral ring scotoma which enlarges peripherally and centrally as the disease progresses.

In the majority of cases, RP is an isolated disorder, but infrequently is associated with other systemic conditions for which treatment strategies have been implicated; for example, abetalipoproteinemia (MIM no. 200100) and Refsum disease (MIM no. 266500). Adult-onset Refsum disease is an autosomal recessive disorder of lipid metabolism caused by a deficiency of phytanic acid hydroxylase. Clinically, patients present in early childhood with cardiomyopathy, ichthyosis, neurologic diseases (polyneuritis, spinocerebellar ataxia, hearing loss, and loss of smell), and odd-shaped red blood cells. The ocular findings include: nystagmus, strabismus, pupillary abnormalities, cataract, and RP. Treatment requires dietary restriction of plant foods and milk which are sources of phytanic acid.

Abetalipoproteinemia or Bassen-Kornzweig syndrome (MIM no. 200100) is an autosomal recessive disorder in which there is abnormal absorption of fat and fat-soluble vitamins, A, D, E, and K. The signs and symptoms of abetalipoproteinemia appear in the first few months of life with failure to thrive, steatorrhea and acanthocytosis. Vitamin A deficiency may result and lead to retinal degeneration that is treatable with vitamin supplementation.

Leber congenital amaurosis (LCA), (MIM no. 204000) was first described in 1869 by Theodore Leber as a congenital form of RP [3, 4]. LCA is an autosomal recessive disorder that is genetically and clinically heterogeneous. LCA is the most severe inherited retinopathy and the most common cause of congenital blindness in children, accounting for 10%–18% of cases [3, 5, 6]. LCA has several phenotypes; symptoms or fundus findings within the first year of life may suggest a particular genotype [3, 7, 8]. Clinical features include: nyctalopia, photoaversion, eye poking (oculodigital sign), nystagmus, hyperopia, an abnormal fundus, and an abnormal ERG [3, 7].

At least 14 genes are associated with LCA and involve various pathways including: retinal development (*CRB1* and* CRX*), phototransduction, (*GUCY2D* and *AIPL1*), vitamin A metabolism (*RPE65*, *LRAT*, and *RDH12*), protein transport (*TULP1*, *RPGRIP1*, and *CEP290*), and RPE phagocytosis (*MERTK*) [8]. Together LCA and juvenile-onset retinal degeneration constitute 70% of cases of severe retinal degeneration or retinal dystrophy. Several of these genes have also been implicated in nonsyndromic or syndromic retinal diseases such as RP and Joubert syndrome, respectively. *CEP290 *(15%), *GUCY2D* (12%), and *CRB1* (10%) are the most frequently genes found to be mutated in cases of LCA.

## 2. Gene Therapy

Gene therapy holds promise for a wide variety of inherited human disease. To date, ocular gene therapy (OGT) has been tried with success in mice, dogs, and now in some humans. OGT requires genetic modification of mutant ocular cells to produce a therapeutic effect. Retinal diseases are excellent targets of OGT as in many cases, the genetic etiology is understood, and there is access to the photoreceptors or the retinal pigment epithelium (RPE) by subretinal injection. In addition, both transgenic and knockout animal models are available that provide preclinical evidence of safety and efficacy. OGT requires first identifying the genetic cause of the RP, and then genotyping patients for mutations in that gene prior to enrolment in gene therapy trials.

Gene therapy strategies differ greatly depending on the inheritance of the disease or more accurately the type of mutation targeted. Some forms of RP are due to loss-of-function mutations (usually autosomal and X-linked recessive). For OGT to be effective, the therapy must replace the missing or insufficient gene product. For example, Tan and colleagues used adenoviral vectors to transduce two mouse models of RP/LCA due to aryl hydrocarbon receptor protein-like 1 (*Aipl1*) deficiency (hypomorphic mutant) and absence (null mutant), establishing the potential of gene replacement therapy in the human condition [9].

Human OGT is most advanced for the form of LCA associated with mutations in* RPE65* [10–14]. Preliminary studies in the Briard dog, a naturally occurring model of LCA (*rpe65*-/-), helped make clinical trials possible. A similar degeneration is seen in the Swedish-Briard/Briard-beagle due to a 4-base pair deletion in the *rpe65 *gene [15]. The initial study of OGT in dogs was done in the USA [5, 6, 16] and later in France [17]. Surgical delivery of recombinant adenovirus associated vectors (AAV) carrying the wild type *rpe65 *cDNA into the subretinal space of three affected dogs demonstrated efficacy as measured by improved ERG responses. The dogs' vision improved in the treated eye and has been stable after five years. More than fifty dogs have since been tested for their response to OGT. Successful gene therapy has also been demonstrated in mice with mutations in the *rpe65* gene [16]. The treatment rescued photoreceptors and also retinal function as measured by the ERG.

The results of separate human trials in the USA, UK, and Italy enrolling patients with mutations in the *RPE65* gene have been reported with encouraging results [11–14]. Bainbridge et al. [11] and Maguire et al. [14] first described separate clinical trials investigating the short-term safety and preliminary efficacy of OGT for LCA in humans. Both groups initially presented short-term data (12 and 5 months, resp.) on three LCA patients enrolled in trials of recombinant AAV delivery of the human* RPE65* gene into the subretinal space. In both studies, patients had severe vision loss documented by visual acuity testing and the ERG. Both studies showed some improvement in navigational testing in at least one patient. This outcome measure has yet to be accepted as a measure of functional visual improvement.

Bainbridge and colleagues studied their patients with microperimetry (which measured retinal sensitivity at precise locations in light-adapted conditions) and observed an improvement after gene therapy in one patient [11]. Maguire et al. observed visual field improvement using Goldmann perimetry and decreased nystagmus after treatment in all their three patients [14] whereas Bainbridge et al. only noted improvement in the dark-adapted perimetry of one patient [11]. Bainbridge et al. [11] showed no change in patients' visual acuity whereas Maguire et al. [14] recorded a gain in visual acuity in all three patients in their study. These outcomes must be replicated with additional subjects and patients' function assessed long term. Further, if safety can be demonstrated, patients with better visual function at baseline should be included in future trials.

Maguire et al. employed the pupillary light reflex as an objective measure of retinal function and found improvement in each of the treated eyes [14]. The pupillary light reflex is a consensual response; a light stimulus to either eye will normally cause both pupils to contract. Fundamentally, it is a measure of the amount of signal input from the photoreceptors, interneurons, and ganglion cells, conveyed through an afferent arc to the brain, with the output driving bilateral pupil constriction. The pupillary response of patients with LCA is significantly diminished, consistent with decreased photoreceptor input to the afferent arc of the reflex [14, 18–20]. In a report of a total of 12 patients (age 8–44) who had undergone OGT for LCA, all had an improvement in the pupillary response, with the greatest effect seen in children [21].

Optical coherence tomography (OCT) allows a noninvasive measure of photoreceptor layer thickness in the central retina of LCA patients [22]. The topography of the photoreceptor layer based on OCT scans, with superimposed retinal landmarks, should be available to the retinal surgeon to guide the subretinal injection of AAV gene vectors. The response to treatment may also be measured with OCT. Photoreceptor loss in the fovea and extrafoveal retina has been shown to be prominent, even in the youngest LCA patient studied. As disease severity in LCA has a broad spectrum, detailed retinal imaging and mapping with OCT should be conducted in all candidates for LCA*-RPE 65 *clinical trials, independent of age [23].

The ERG responses were extremely low or undetectable in patients in both studies at baseline and remained unchanged after treatment. Whether the improvements in retinal function are reproducible and persistent in subjects remain as questions along with whether retinal degeneration is delayed or averted. Systemic or ocular complications may yet be encountered as additional patients are treated with higher doses of vector and followed for longer periods. 

Alternates to OGT for the treatment of LCA are also being pursued; for example, oral administration of a retinoid, QLT091001 (NCT00765427, NCT01014052, see: http://www.clinicaltrials.gov/). Preliminary results, presented at the Association for Research on Vision in Ophthalmology meeting in May, 2010, suggested improved function in three LCA patients with *RPE65* and *LRAT* mutations.

For autosomal dominant RP, caused by gain-of-function mutations, effective therapy must either prevent the mutant protein from being produced or counter the expression of the protein. Ribozymes catalyze enzymatic reactions that break down RNA [24, 25]. Conceptually, it would, therefore, be possible to use ribozymes to treat autosomal dominant RP by blocking the gene product from the mutant allele, thereby halting or slowing the progression of the disease. In 1998, Drenser et al. [26] showed that ribozyme could be used to decrease the amount of mutant rhodopsin messenger RNA. Later, the same group used recombinant AAV to transduce photoreceptor cells of rhodopsin mutant (pro23his) transgenic rats with ribozyme and an opsin promoter, demonstrating that ribozyme could slow photoreceptor degeneration. They showed that treatment was effective at age 1 month and 1.5 months when 40%–45% of photoreceptors would have normally degenerated [27, 28]. The pro23his mutation in rhodopsin represents a change from proline to histidine at position 23 and is the most common rhodopsin mutation in humans. By targeting only the mutant RNA sequence, ribozyme therapy is mutation-dependent and therefore limited in its application. Autosomal dominant RP is genetically heterogeneous; 25% of cases are caused by different mutations in rhodopsin and the remaining cases are not linked to rhodopsin. Unique gene therapies with a large number of ribozymes would have to be developed for each of these disorders.

RNA interference (RNAi) is mutation-independent and a powerful method for posttranslational gene silencing. In mammalian systems, small interfering RNAs (siRNAs) are introduced directly into the cell or processed in the cell from translated short hairpin RNA (shRNA) [29] and then assembled into an RNA-induced silencing complex known as RISC. RISC allows the antisense strand to form a duplex with the target messenger RNA which is then degraded by an enzyme, and then rendered inactive. Compared to ribozyme therapy, RNAi is at least as potent, less dependent on RNA secondary structure and does not require a particular sequence motif. RNAi has been used to identify genes that promote apoptosis or oxidative damage in retinal cells and could provide new avenues for treatment of photoreceptor degenerations [30, 31].

## 3. Retinal Implants

The treatment of RP patients with severe visual loss using either epiretinal or subretinal implants was reviewed recently by Margalit et al. [32]. Humayun et al. reported direct retinal stimulation using epiretinal implants in RP patients [33]. Using a 16-electrode array, patients saw spots of light that were usually colored (yellow/blue/yellow-green) and the direction of movement (http://www.artificialretina.energy.gov/ and Second Sight Medical Products, Inc. Sylmar, CA). Resolution in this model is believed to be up to 1.8 degrees of visual field. At the 2009 annual meeting of the Association for Research on Vision in Ophthalmology, the Artificial Retina Project released an update on the Argus II, a 60-electrode retinal prosthesis. As of March 31, 2009, 21 people with RP had been implanted with the device; this number continues to rise as more subjects are enrolled in a Phase II, three year clinical trial. Although the Argus II prosthesis consists of an array of 60 electrodes attached to the retina, the project aims to increase the number of electrodes beyond 200.

Caspi et al. [34] used a 16 electrode retinal prosthesis in a totally blind subject with RP. The implant was controlled wirelessly by an external computer and head mounted video camera. Spatial vision was assessed by measuring the subject's response to direct stimulation patterns and by comparing the ability of the subject to identify the orientation of gratings with the system on and off. Results showed that synchronized stimulation of different retinal locations could produce spatial vision long term with an acuity level determined by the distance between the electrodes.

Yanai et al. [35] assessed visual task performance in three subjects blinded by RP. An epiretinal prosthesis was implanted in the eye with worse vision and the input was wirelessly controlled by a computer or head-worn video camera. Subjects scored better in 8 of 9 computer-controlled experiments. This study, although small in size, suggested that a low-resolution, epiretinal prosthesis could provide visual information to perform simple tasks that were impossible with only light perception vision.

Subretinal electrodes have been attempted in animal models and the results indicate that cortical activity can be induced [36, 37]. Similar experiments have since been initiated in humans [38]. The long-term effect of the implants has not been assessed, nor has the effect of the electrodes placed between the neuroretina and the retinal pigment epithelium on retinal metabolic function.

## 4. Neurotrophic Factors

Several neuorotrophic factors have been shown to protect photoreceptors from degeneration, including ciliary neurotrophic factor (CNTF). In different animal models of retinal degeneration, CNTF was shown to delay photoreceptor degeneration [27, 39–42]. A thicker outer nuclear layer was observed in treated animals, reflecting preservation of the photoreceptors and anatomical rescue. Electrophysiological recordings performed to evaluate retinal function demonstrated an improvement in the scotopic and photopic responses recorded from CNTF-treated eyes compared to untreated eyes [39, 41]. Glial cell line-derived neurotrophic factor (GDNF) has also been shown to have a neuroprotective effect on degenerating photoreceptors by slowing down the degeneration of rods while preserving visual function [42]. 

While neuroprotective factors may offer promising results in the treatment of RP in animal models, effective treatment strategies need to be developed for clinical delivery. Direct intravitreal or subretinal neurotrophic factor injections have been performed in animal models with therapeutic effect; [43] however, an implantable device allows for long term delivery avoiding repeated injections with the risk of mechanical or infectious complications.

*Ex vivo* gene therapy is a promising approach whereby genetically engineered and encapsulated human retinal pigment epithelial cells are implanted into the vitreous in a device [44]. A Phase I safety trial of the delivery of CNTF through encapsulated cell therapy was completed on patients with RP (one of which had choroideremia) without serious adverse event and some suggestion of improvement in visual acuity. A Phase II trial are currently underway and designed to show efficacy in treating atrophic macular degeneration and RP [45].

## 5. Retinal Transplantation

Retinal transplantation places sheets of developing retina and retinal pigment epithelial cells into the subretinal space [46]. Whereas adult transplants have been performed in humans with RP and age-related macular degeneration (AMD); [47] the transplants have not caused harm but there is no evidence that the cells of the transplanted tissue mingle with or develop synaptic connections. Radtke and his group reported efficacy and safety in implanting fetal retina with accompanying RPE in AMD and RP patients with vision of 20/200. Seven of the ten patients showed improved visual acuity, corroborating results in animal models of retinal degeneration [48].

An alternate approach may be the transplantation of photoreceptor precursors. MacLaren and colleagues demonstrated that the timing of the harvest of the donor cells must be at the correct stage of rod morphogenesis, when they have exited the cell cycle and are in the first stages towards becoming mature photoreceptors [49]. If the cells were isolated just a couple of days too early or too late, they would not integrate into the retina. When successful, the treated eye showed an improved pupillary light response suggesting that the transplanted cells were responsive to light and had integrated into the retinal circuitry connecting to the central nervous system.

Lamba et al. incubated human embryonic stem cells in a complex cocktail that coaxed cells into becoming photoreceptor progenitors [50]. These progenitors, like the *in vivo* derived progenitors described by MacLaren et al., were able to integrate into degenerated mouse retinas [49].

It may be possible to prepare unlimited numbers of progenitor cells that are suitable for transplantation regardless of whether donor progenitor cells are isolated from adult tissue or from embryonic stem cells. How can one ensure a sufficient number of stem cells that are available for an effective graft? MacLaren et al. showed that it is not necessary to integrate each precursor cell with each secondary neuron to achieve a therapeutic effect [49]. Also, it may not be necessary to treat the entire retina; treatment of the macula alone may suffice.

## 6. Stem Cells

Enzmann and colleagues have reviewed the use of stem cells, their plasticity, their ability to give rise to specialized cells, and their capacity for self-renewal [51]. Lund and coworkers have derived RPE cells that are critical to the health of photoreceptors from human embryonic stem cells [52]. The RPE cells were then transplanted into rats with retinal degenerative disease. The investigators reported that the improvement in vision of treated rats was 100% over untreated controls. Although the RPE cells were not sufficiently developed to completely replace the damaged RPE, they were able to rescue vision by the long-term production of growth factors beneficial to the health of the retina. Lund and his collaborators are proceeding to produce entirely functional RPE and photoreceptors from stem cells to replace and repair degenerated retinas in humans.

## 7. Light Protection

Clinical evidence and data from animal studies suggest that some pigmentary retinopathies are particularly susceptible to light damage [53]. Patients with RP are advised to wear dark glasses outdoors. The use of amber spectacles should block ultraviolet rays and visible wavelengths up to about 527 nm. Outdoors, it is ideal to use spectacles that block ultraviolet rays and light up to approximately 550 nm to filter blue light.

## 8. Vitamin Therapy

Vitamin A may protect the photoreceptors by trophic and antioxidant effects. Long-term (5 to 15 year) vitamin A supplementation in doses of 15,000 IU per day slowed down the loss of ERG amplitudes [54]. Vitamin E at 4,000 IU had an adverse effect [54]. Clinicians continue to debate the conclusions of these studies [55]. There is no consensus about the utility of vitamin A treatment. Vitamin A should not be given to patients with RP caused by mutations in the *ABCA4* gene. In another study, RP patients were given docosahexaenoic acid (DHA) supplementation at 1200 mg/day in addition to vitamin A [56]. This study showed that the disease course was initially slowed by the addition of DHA; however, the beneficial effect did not last beyond two years. Berson and colleagues have reported on the benefits to RP patients of a diet rich in omega-3 fatty acids [57]. RP patients taking vitamin A palmitate, but not DHA capsules, benefited from an omega-3 rich diet (equivalent to eating salmon, tuna, mackerel, herring, or sardines, once to two times a week). Recently, Berson and colleagues reported on patients taking Vitamin A randomly assigned to either lutein supplementation (12 mg/da) or placebo over a four year period [58]. Lutein appeared to slow the decline in the mean rate of sensitivity loss as measured by the Humphrey visual field 60-test. An accompanying article in the same journal discussed carefully the merits of all these studies [59]. 

A study of the potential benefit of DHA in patients with X-linked RP is ongoing [60]. Patients between the ages of 8 and 32 who have X-linked RP are enrolled in a four year, Phase II, clinical trial studying the effect of nutritional supplementation with DHA. DHA is a component of cell membranes throughout the body, and most highly concentrated in the retina and the brain where it plays a role in phototransduction and synaptic transmission [47].

## 9. Drug Delivery

A group of international experts in drug delivery are studying the treatment of retinal degenerative diseases by long-term, sustained drug delivery through the sclera [61–64]. They are also investigating a range of delivery devices such as microneedles, collagen gels, and the use of an electric field. Better methods of drug delivery could be crucial for future therapies to save or restore sight.

## 10. Macular Edema

Cystoid macular edema (CME), which occurs frequently in RP patients, is often chronic and may not improve with carbonic anhydrase inhibitors [65, 66]. A trial of therapy over two months may instituted, and if effective, should be continued indefinitely; if no response is seen with treatment, it should be discontinued. Care must be taken when considering long-term acetazolamide as it has been shown to depress the ERG responses in mice [67]. In a small trial of 20 treated RP patients and 20 matched untreated RP controls, intravitreal triamcinolone (4 mg) did not result in a statistically significant improvement in best corrected visual acuity [68].

## 11. Conclusion

Therapies are becoming available to restore vision or stop the progressive loss of visual function caused by pigmentary retinopathies. The psychological boost to researchers, patients, and families from the results of LCA gene therapy trials is very evident. Therapeutic strategies are being designed and applied to slow down the degenerative process, to treat ocular complications, and to help with the social and psychological impact of blindness resulting from RP. Approaches to therapy for RP now include: gene therapy, neurotrophic growth factors, anti-apoptotic agents, ribozyme therapy, RNAi, retinal transplantation, dietary supplementation, retinal prostheses, and stem cell therapy. We hope that, in the future, discoveries from the laboratory will be brought into the clinical setting.

## 12. Method of Literature Search

References for this paper were identified through a comprehensive English-language literature search of the electronic Medline database (1993–2009), using the Medline search service. Search of other databases did not add to the search of Medline. The following key words were used alone or in combination: retinitis pigmentosa, rod-cone dystrophy, RP, RNAi, neurotrophic growth factors, encapsulated cellular therapy, ciliary neurotrophic factor, anti-apoptosis, genes, bionic eye, precursor photoreceptor transplantation, optical aids, cystoid macular edema, Leber congenital amaurosis, retinal cell transplantation, precursor photoreceptors, treatment, and Vitamin A. 

##  Conflict of Interests

The authors have no proprietary or commercial interest in any product mentioned or perhaps discussed in this paper.

##  Websites

1. Artificial Retina Project, US Department of Energy Office of Science. http://www.artificialretina.energy.gov/
2. Daiger, S. RetNet. http://www.sph.uth.tmc.edu/retnet/sum-dis.htm/
3. Clinical trials: http://www.clinicaltrials.gov/
